# Survey of UK pet owners quantifying internal parasite infection risk and deworming recommendation implications

**DOI:** 10.1186/s13071-020-04086-2

**Published:** 2020-04-26

**Authors:** Christopher Pennelegion, Jason Drake, Scott Wiseman, Ian Wright

**Affiliations:** 1 Elanco Animal Health, Lilly House, Priestley Road, Basingstoke, Hampshire RG24 9NL UK; 2 Elanco Animal Health, 2500 Innovation Way, Greenfield, IN 46140 USA; 3 Mount Veterinary Practice, 1 Harris St, Fleetwood, FY7 6QX UK; 4 ESCCAP UK & Ireland, PO Box 358, Malvern, WR14 9HQ UK

**Keywords:** ESSCAP, Tapeworm, Risk assessment, Cestode, Endoparasites, Zoonosis, *Toxocara*, Dogs, Cats, Nematode, Parasite control

## Abstract

**Background:**

Dogs and cats in the UK are exposed to many internal parasites which can pose risks to the health of both the pet and their owners. By understanding these endemic parasites and the risks they pose, we can assess the lifestyle of pets and recommend the correct deworming frequency. Studies identifying risk factors were discussed in the European Scientific Counsel Companion Animal Parasites (ESCCAP) guidelines. To this date, there has been very little information on how pet owners in the UK deworm their pets and if the protocols they follow align with ESCCAP recommendations. The objective of this study was to look at the current deworming protocols of UK cat and dog owners in conjunction with their lifestyle and risk.

**Methods:**

An online survey was conducted in the UK targeting pet owners who own at least one dog and/or cat and were responsible for product purchase, the pet’s health care and veterinary visits. These survey results were analysed against the ESCCAP guidelines and each pet placed into a risk category. By comparing the current deworming frequency with that recommended for their risk category, the compliance of UK pet owners with ESCCAP recommendations was evaluated.

**Results:**

A total of 500 dog owners and 500 cat owners completed surveys. Overall, the study found none of the pets fell into risk group A, with all pets meeting the risk level for at least deworming four times a year (risk group B and above). The majority of animals fell into the highest risk category D with 97% of dogs and 68% of cats. The average deworming per year in the UK was 3.1 for dogs and 3.1 cats, below the minimum recommended by ESCCAP.

**Conclusions:**

For both felines and canines, the dosing frequencies are lower than recommended to both reduce zoonotic risk for reducing *Toxocara* spp. egg-shedding and improve pet health. This research highlights the need for improved education around dog and cat patient risk assessments and greater adherence to recommended deworming aligned with the ESCCAP guidelines.
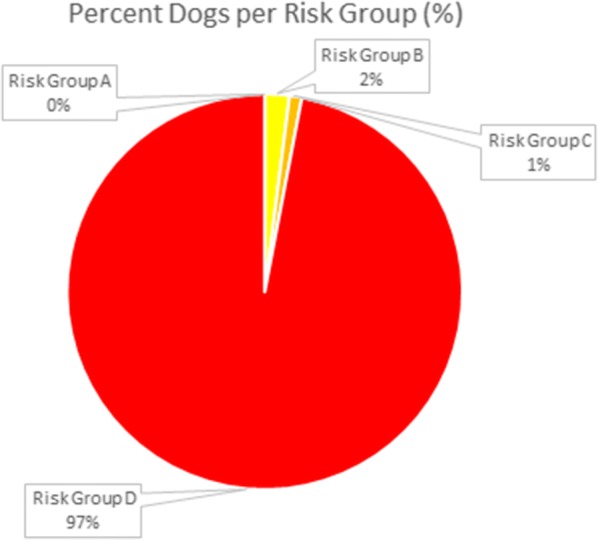

## Background

A survey was conducted across five European countries which collected parasite exposure and infection risk assessment information from 5001 pet owners [1]. This manuscript delves more deeply into the 1000 pet owner responses from the UK from this previous survey.

In the UK, there are a number of internal parasites commonly found in cats and dogs. Alongside the potential adverse effects on the health of the pet, these endoparasites can impact the health of livestock and pose a potential zoonotic threat to human health [2]. By understanding the epidemiology of these parasites, we can suggest appropriate deworming frequencies for our pets based on their personal risk and the risk they pose to the families they live with [2].

Many endoparasites are present in the UK, but three main groups are of major concern; tapeworms, roundworms and lungworms [2]. The three tapeworms of concern are *Echinococcus granulosus*, *Taenia* spp. and *Dipylidium caninum*. Most often these endoparasites do not cause any harm to our pets, but tapeworm segments are visible to pet owners. *Dipylidium caninum* is contracted from ingesting infected fleas, producing large motile proglottids in faeces or emerging from the anus of infected animals. *Taenia* spp. have occasionally been reported to cause intestinal blockages [3], but generally infection goes unnoticed. However, *Taenia* spp. such as *Taenia ovis* and *Taenia hydatigena* proglottids spread by pets on pasture, contribute to major economic loss in excess of £5 million annually in the UK *via* ruminant offal and carcass condemnation [4]. *Echinococcus granulosus* infection is subclinical in dogs but is responsible for a severe and potentially life-threatening disease if infectious eggs shed from the dog are accidently ingested by humans. While *D. caninum* and *Taenia* spp. are ubiquitous over the UK, *E. granulosus* has historically been an issue for Wales and the Western Isles of Scotland. However, recent work looking at potential infections from carcases infected at abattoirs showed a much wider spread than expected [5]. A long incubation period of many years in humans means that there would also be a significant delay before an increase in UK incidence of human hydatid infection was recognised [5].

The most common roundworms in dogs and cats, respectively, are *Toxocara canis* and *Toxocara cati*. Most veterinary surgeons are aware of their zoonotic potential and there is some public knowledge of their effects in people, especially children [6, 7]. As well as the recognised syndromes of ocular and visceral toxocarosis, meta-analyses have concluded people seropositive for *Toxocara* spp. are also more likely to be associated with asthma, epilepsy and learning difficulties [8–11]. They are often overlooked in adult pets as a risk factor or potential zoonosis, yet these are the most prevalent endoparasites in adult dogs and cats in the UK [12]. No recent data exist regarding the epidemiology across the whole of the UK; however, regional studies have been carried out. A study in Lancashire in 2016 showed that 5.3% of dogs and 26% of cats tested by fecal flotation were actively excreting *Toxocara* eggs in their faeces [13]. Another study conducted in Bristol in 2013 used local data and a predictive model to look at the main species that contribute to *Toxocara* spp. eggs in the environment. It concluded that out of dogs, cats and foxes, the main contributors to contamination of the environment were owned dogs [14].

Lungworm in the UK is highly publicised, with the most common worm treated and prevented for being *Angiostrongylus vasorum*. Its potential severe health effects on dogs has gained it notoriety among both veterinary professionals and the general public. The first case in the UK was reported in the South West in 1980 [15]. Studies looking at the presence of *A. vasorum* in the fox, its reservoir host, showed that that prevalence of infection in 2008 was 7.3% nationally, with a heavy skew towards the South East of 23% with 0% in the North and Scotland [16]. In 2015, the national average jumped to 18.3% with significant increases in most areas of the UK [17].

When looking at these endoparasites commonplace in the UK, the risks posed to the pet and owner must be considered. These implications were summarised by the European Scientific Counsel Companion Animal Parasites (ESCCAP), then further refined for our geography by ESCCAP UK and Ireland. ESCCAP uses research-based independent advice available to form these assessments, provides education to veterinarians and pet owners and suggests appropriate ‘risk based’ deworming advice. The main recommendations appropriate to this study can be found within their document ‘Worm control in dogs and cats’ [2].

UK pet owners deworm currently for a mixture of pet health and to prevent zoonosis [6, 7, 18]. However, the frequency at which this is carried out likely falls below what is recommended. Pet owners often only treat for worms that have consequences (e.g. *A. vasorum*), but being blind to the presence of other worms harms overall compliance and frequency [12].

ESCCAP UK and Ireland recommends deworming no less than four times a year, with certain risk groups requiring more frequent deworming, up to monthly. This is based on a pet’s personal risk, taking into account factors such as freedom to roam, eating carrion or actively hunting. Additional considerations include pregnancy/lactation status, the animal’s age (e.g. deworming of puppies is needed more frequently), eating slugs/snails, travel to certain areas (e.g. areas with endemic *Echinococcus* spp.), and contact with children or immunocompromised individuals.

The primary objective of this study is to describe the deworming frequency of dog and cat pets from the UK and how they fall into the mutually exclusive ESCCAP parasite risk categories according to their lifestyle. The secondary objective is to analyse deworming practices in these dogs and cats and assess how that frequency conforms to ESCCAP deworming guidelines.

Research questions to be answered: What percentage of cats and dogs has a specific risk/lifestyle which increases the chance of catching worms? How often are cats and dogs with various lifestyles dewormed? With regard to their geographical location and lifestyle, how often should cats and dogs be dewormed?

## Methods

The methods of this survey have been described previously [1]. The following provides a short summary of the methods.

### Design, setting and sample

An online survey was conducted in the UK from the 3rd July 2017 to the 14th July 2017; targeting pet owners who own at least one dog and/or cat and were responsible for product purchase, the pet’s health care and veterinary visits.

A custom online panel of pet owners was used to recruit a target sample of *n* = 500 cat owners and *n* = 500 dog owners. The survey was confidential and anonymous, with respondents being offered a small incentive for completion.

Respondents had to own at least one dog and/or cat and be at least 18 years of age in order to participate. If the household contained both cats and dogs, participants were assigned randomly to either the dog or the cat group. Owners had to be responsible for veterinary visits, product purchases and the pet’s health and share or have sole responsibility for the dog or cat. Both cats and dogs had to have been seen once a year by a veterinary surgeon. Owners with more than 10 cats or dogs, breeders and traders were excluded. UK demographic statistics using data from the Office for National Statistics [19] and proprietary research data as described in [1] about UK dog and cat owners were used to set quotas. These quotas related to region, age and sex, employment status, household size (including number of children present in the household).

The main survey contained nine questions about the current deworming protocol and the cats’ and dogs’ lifestyle. Differing questions were utilized for dog and cat owners to align with the ESCCAP guidelines.

The ESCCAP parasite infection risk assessment guidelines were used to create the survey questions. The pet owner responses regarding exposure risks and pet behaviour placed the pet into one of four distinct risk groups, as defined in Table 1 (dogs) and in Table 2 (cats).

**Table 1:** Dog risk group definitions

Dog risk group	Description	EU ESCCAP recommended deworming frequency
A	Older than 6 months, lives indoors only or goes outdoors but has no direct contact with parks, sandpits, playgrounds, and (faeces from) other dogs and cats, snails and slugs, raw meat or prey	1–2 times per year
B	Older than 6 months, goes outdoors and has direct contact with parks, sandpits, playgrounds, and (faeces from) other dogs and cats; but does not eat prey animals and/or snails and slugs and/or goes outdoors to hunt and does not eat raw meat	4 times per year
C	Older than 6 months, goes outdoors and has direct contact with parks, sandpits, playgrounds, and (faeces from) other dogs and cats and eats prey animals and/or snails and slugs and/or goes outdoors to hunt and eats raw meat	> 4 times per year
D	Is less than 6 month-old; or lives in a fox tapeworm (*Echinococcus multilocularis*) endemic area; or eats prey animals and/or goes outdoors to hunt; or lives indoors, eats raw meat and lives with children/elderly	Monthly

**Table 2:** Cat risk group definitions

Cat risk group	Description	EU ESCCAP recommended deworming frequency
A	Cat lives indoors. Infection pressure with worm stages is low, eating rodents unlikely	Treat 1–2 times per year against roundworms, or 1–2 times per year fecal exam and treatment according to findings
B	Cat is free to roam outdoors. Infection pressure with worm stages is high, eating rodents likely	Treat against roundworms and tapeworms at least 4 times a year
C^a^	Cat eats prey animals and/or goes outdoors to hunt and eats raw meat	More than 4 times per year
D^a^	Cat is free to roam outdoors and shares home with young children or immunocompromised individuals	Deworm once a month, or examine faecal samples once a month and treat according to findings

^a^ESCCAP Cat Risk Groups include A and B only. Additional risk factors in the ESCCAP guidelines were used to create Groups C and D for consistency in reporting and comparison of dog and cat results

### Statistical methods

The association between the frequency of deworming and risk group was investigated by constructing a contingency table of risk group against frequency of deworming and testing the null hypothesis of no association between the variables using Cochran–Mantel–Haenszel (CMH) test statistics.

The survey questions were based upon both individual country ESCCAP and EU ESCCAP guidelines which are designed for assessing parasite infection risk [2, 19–21]. The responses from pet owners regarding exposure risks and pet behaviour were used to place the pet into 1 of 4 distinct risk groups, as defined in Table 1 (dog risk group definitions) and Table 2 (cat risk group definitions). Although the EU ESCCAP guidelines only have two risk groups for cats (A and B), the ESCCAP guidelines provide additional risk factors outlined in a table labelled “additional treatments for cats” [2]. This table shows a need for monthly deworming of cats which are in close contact with immunosuppressed individuals or young children, and recommends deworming 4–6 times a year for cats which are not closely supervised. These factors along with feeding of raw diets were then used to formulate two additional feline risk groups (C and D). The “additional treatments for dogs” recommends dogs in close contact with immune compromised people or young children be dewormed monthly, so these dogs were also then included in risk group D. The proportion of pets, according to risk group, which were aligned to deworming recommendations was estimated. Pet owners were considered aligned if they meet or exceed the deworming frequency required for their risk group identified by the survey.

With these alignments made, the survey results could be consistently reported for both dogs and cats, while also acknowledging the guideline conditions where monthly deworming for cats is indicated.

## Results

Surveys were completed by 500 cat and 500 dog owners, respectively, in the UK. A total number of 22,890 people were invited with 2312 opening the invitation link. Of those, 93 participants did not complete the survey, 921 were excluded as they did not meet the target criteria and 298 were quota out. “Quota out” were participants who attempted to complete the survey after the quota of 500 dog owners or 500 cat owners was met.

For dogs, 98.4% owned by survey participants were greater than 6 months of age. The potential risk factors most commonly identified in descending order were: goes beyond the garden (89.4%); contact with other animals (81.6%); and interacts with children or the elderly (80.6%) (Table 3). This study did not give a strict definition of “children” and “elderly” and so the 80.6% figure may be an overestimate. When looking across the 12 different regions surveyed in the UK, the trend in risk is similar apart from Northern Ireland, but respondents in this area were only 3 (Table 3). Looking at all the risk factors combined the most common lifestyle of dogs in the UK was off the lead and lives with children with 28.4% of the dog owners surveyed (Table 4).

**Table 3:** Results from canine lifestyle survey answers (in %) and deworming frequency per region in the UK

	East Midlands (*n* = 51)	Eastern (*n* = 25)	London (*n* = = 21)	North-East (*n* = 37)	North- West^a^ (*n* = 57)	N. Ireland (*n* = 3)	Scotland (*n* = 43)	South-East (*n* = 76)	South-West (*n* = 52)	Wales (*n* = 41)	West Midlands (*n* = 51)	Yorkshire^b^ (*n* = 43)	UK average (*n* = 500)
Adult dogs	100	100	95.2	97.3	98.2	66.7	100	98.7	98.1	97.6	98.0	100	98.4
Goes outside beyond garden	84.3	100	90.5	94.6	91.2	66.7	86.0	90.8	78.8	85.4	96.1	98.0	89.4
Catches prey	13.7	12.0	14.3	13.5	10.5	33.3	9.3	17.1	9.6	7.3	13.7	14.0	12.6
Eats raw meat	13.7	20.0	19.0	3.0	5.0	33.3	16.3	9.0	11.5	7.3	9.8	4.7	10.2
Interacts with children/elderly	82.4	80.0	76.2	78.4	86.0	66.7	79.1	82.9	78.8	78.0	78.4	81.4	80.6
Exercising off lead	62.7	56.0	66.7	62.6	63.2	33.3	62.8	73.7	63.5	65.9	72.5	60.5	65.2
Eats slugs/snails/grass	37.3	36.0	33.3	29.7	33.3	66.7	32.6	34.2	34.6	29.3	23.5	37.2	33.0
Contact to other animals	80.4	88.0	76.2	81.1	87.7	66.7	74.4	88.2	80.8	80.5	80.4	74.4	81.6
Average deworming per year	3.2	3.0	2.7	2.8	3.1	4.7	3.0	3.5	3.2	3.2	3.0	3.4	3.2

^a^ Including Merseyside

^b^ Including The Humber

**Table 4:** Canine lifestyle and deworming

Risk group	Percentage of dogs	Most common risk profile	Current average no. of dewormings
Risk Group A	0	na	na
Risk Group B	2	Exercises only in the garden, highly supervised	2
Risk Group C	1	Exercised off the lead	3
Risk Group D	97	Off the lead and lives with children	2.99
		Off the lead, eats slugs/snails/grass and meat (prey or raw meat) and lives with children	3.53

*Abbreviation*: na, Not applicable

**Fig. 1:**
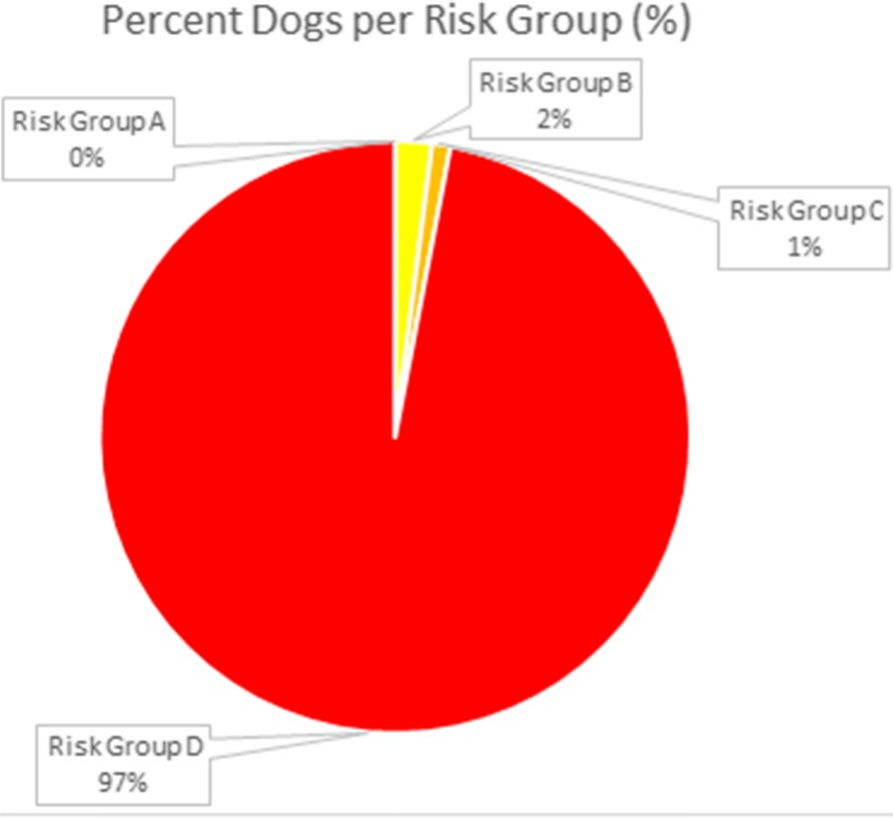
Percent of dogs in each ESCCAP Risk Group

**Fig. 2:**
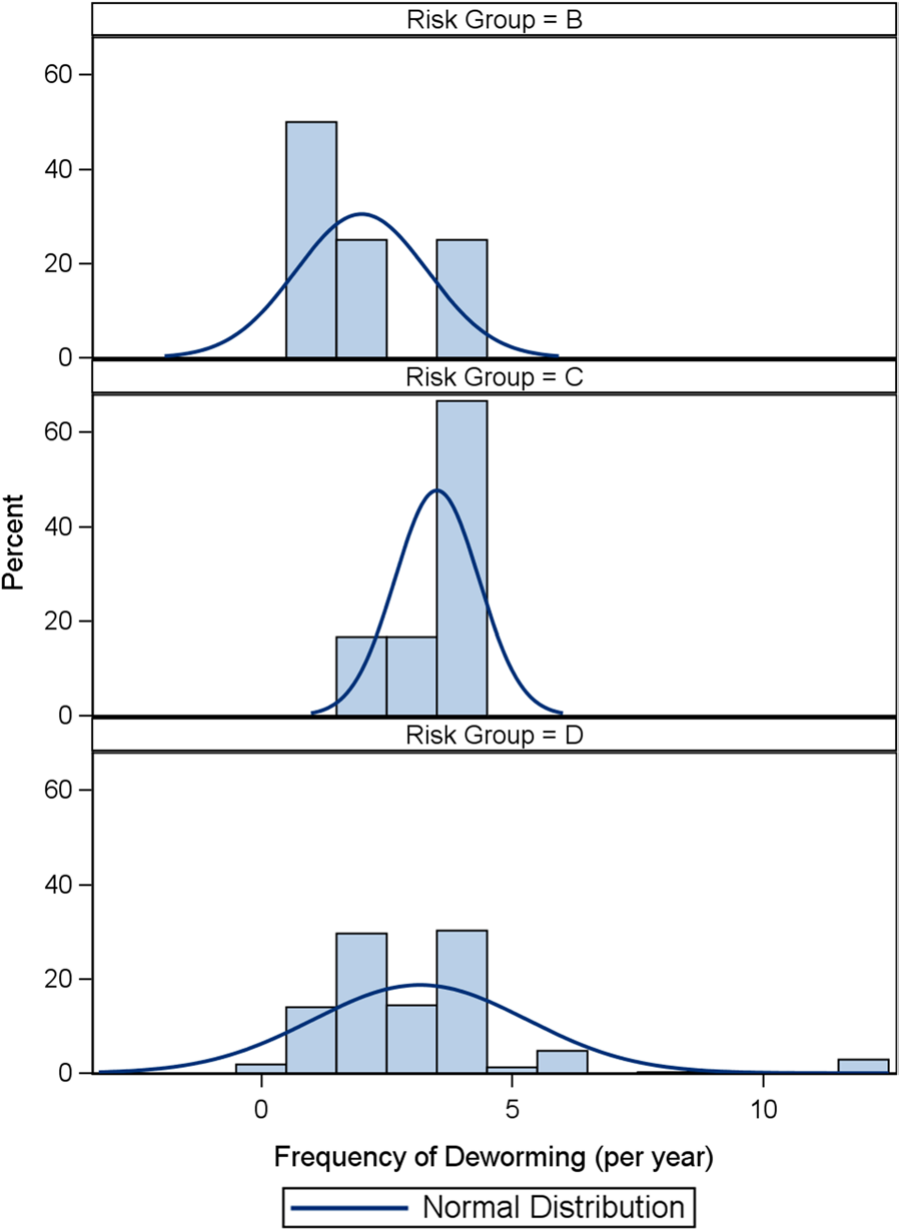
Distribution of deworming frequency of dogs in each ESCCAP Risk Group

Overall in the UK, 97% of dogs fell into ESCCAP risk group D, 1% in C and 2% in B, with none falling into risk group A (Table 4, Fig. 1). However, the average deworming was only 3.1 doses per year. ESCCAP UK and Ireland recommends monthly anthelmintic treatments for group D animals and no less than 4 times per year for any animal, including those in group A. In dogs, the mean frequency of deworming in groups B, C and D were 2, 3.5 and 3.2, respectively (Table 5). The median frequency in groups B, C and D was similar at 1.5, 4 and 3 times per year, respectively, with little to no location shift of the center of the frequency distribution in these statistics (Fig. 2). Despite the low average frequencies in these groups, the proportion of dogs in groups B, C and D considered to be deworming in alignment with ESCCAP recommendations were 25%, 0% and 8.6%, respectively (Table 5). However, due to the number of dewormings required in order to be considered aligned, this may be an overestimate of the actual number in true alignment. Statistical analysis showed no association between risk group and frequency of deworming (QCS = 1.5946, *df* = 1, *P* = 0.2067).

**Table 5:** Canine and feline frequency of deworming and alignment with ESCCAP recommendations

Risk group	Statistic	Canine (*n* = 500)	Feline (*n* = 500)
A	No cases		
B	Mean	2.0	2.4
	Minimum	1	0
	Q1	1	1
	Median	1.5	2
	Q3	3	4
	Maximum	4	12
	% aligned	25.0	37.1
C	Mean	3.5	2.9
	Minimum	2	1
	Q1	3	2
	Median	4	3
	Q3	4	4
	Maximum	4	6
	% aligned	0.0	11.8
D	Mean	3.2	3.4
	Minimum	0	0
	Q1	2	2
	Median	3	3
	Q3	4	4
	Maximum	12	12
	% aligned	8.6	13.8

For cats, 98.2% owned by survey participants were greater than 6 months of age. The potential risk factors most commonly identified in descending order were: goes outdoors (79.8%); hunts (57.6%); and interacts with children or the elderly (51.8%) (Table 6). When looking across the 11 different regions surveyed in the UK, the trend in risk is relatively similar apart from consuming raw meat which seems to vary by region. There is also some variation in the number of cats which are outdoors (Table 6). Looking at all the risk factors combined the most common lifestyle of cats was lives with children, goes outdoors, and hunts/catches prey with 29% of the cat owners surveyed (Table 7).

**Table 6:** Results from feline lifestyle survey answers (in %) and deworming frequency per region in the UK

	East Midlands (*n* = 47)	Eastern (*n* = 58)	London (*n* = 26)	North East(*n* = 13)	North West^a^ (*n* = 64)	Scotland (*n* = 36)	South East (*n* = 90)	South West (*n* = 53)	Wales (*n* = 29)	West Midlands (*n* = 43)	Yorkshire^b^ (*n* = 41)	UK average (*n* = 500)
Adult cats	97.9	98.3	96.2	100	100	100	97.8	96.2	100	97.7	97.6	98.2
Outdoor	89.4	70.7	84.6	84.6	78.1	80.6	84.4	83.0	75.9	76.7	70.7	79.8
Catches prey	59.6	41.4	50.0	53.8	53.1	55.6	48.9	56.6	48.3	46.5	31.7	49.4
Eats raw meat	0	3.4	3.8	0	1.6	5.6	8.9	0	0	7.0	7.3	4.0
Interacts with children/elderly	55.3	50.0	38.5	53.8	54.7	47.2	52.2	56.6	55.2	48.8	54.2	51.8
Hunting	68.1	50.0	57.7	53.8	57.8	66.7	55.6	69.8	55.2	55.8	43.9	57.8
Average deworming per year	3.3	2.7	3.2	3.1	2.7	2.5	3.5	3.6	2.83	3.0	3.1	3.1

^a^ Including Merseyside

^b^ Including The Humber

**Table 7:** Feline lifestyle and deworming

Risk group	Percentage of cats	Most common risk profile	Current average no. of dewormings
Risk group A	0	na	na
Risk group B	29	Cat goes outdoors, but is supervised	2.36
Risk group C	3	Cat goes outdoors, hunts/catches prey	3.21
Risk group D	68	Cat goes outdoors, hunts/catches prey and lives with children	3.37
		Cat goes outdoors, is supervised and lives with children	3.26

*Abbreviation*: na, Not applicable

**Fig. 3:**
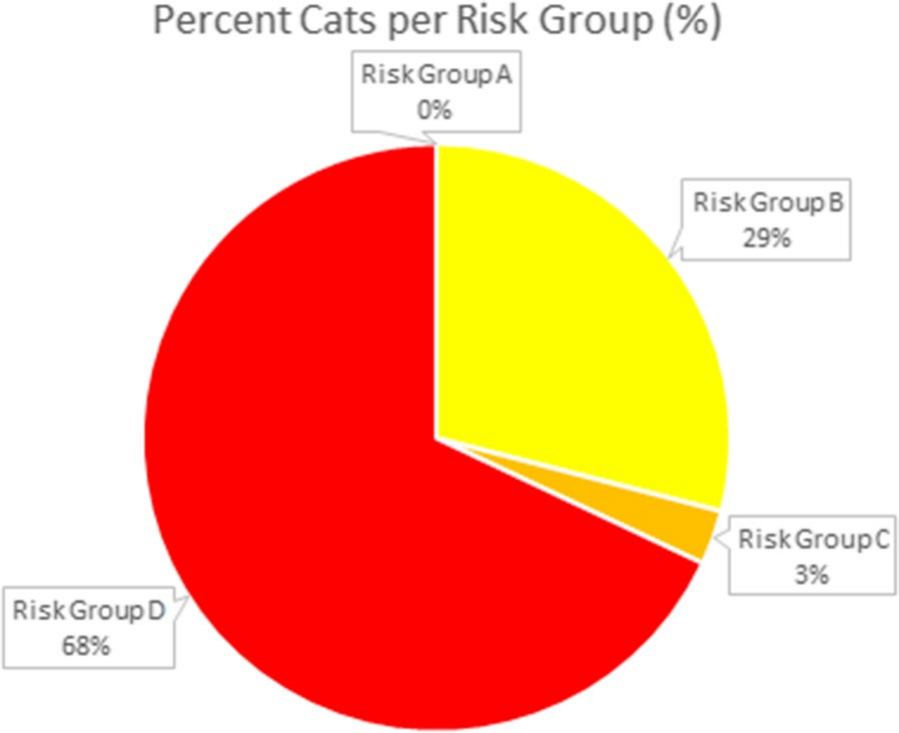
Percent of cats in each ESCCAP Risk Group

**Fig. 4:**
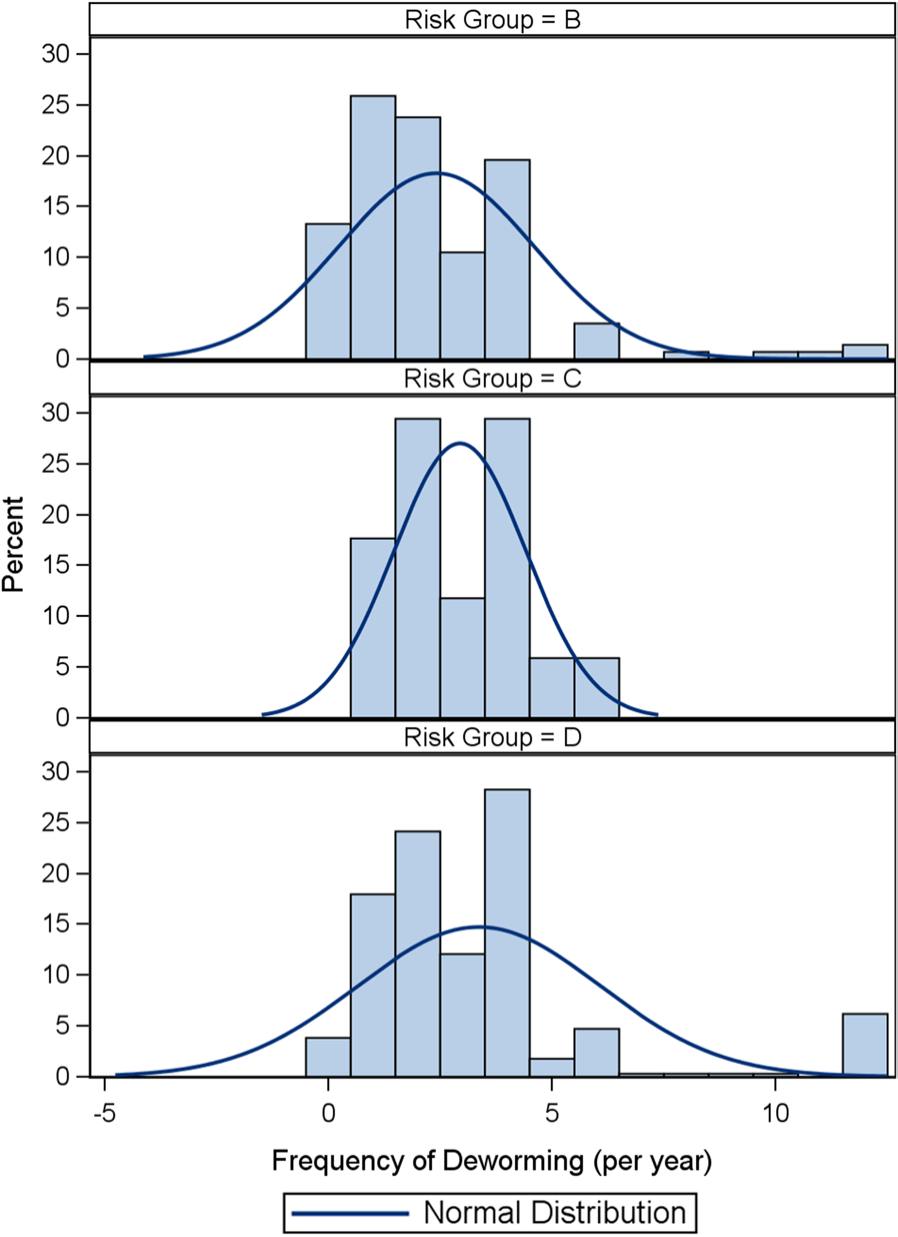
Distribution of deworming frequency of cats in each ESCCAP Risk Group

Overall in the UK, 68% of cats fell into risk group D, 3% in C and 29% in B with none falling into risk group A (Table 7, Fig. 3). However, the average number of dewormings a year for cats was 3.1 doses. The minimum doses recommended for cats in group D would be monthly with all other groups dewormed at least 4 times per year. In cats, the mean frequency of deworming in groups B, C and D were 2.4, 2.9 and 3.4 doses per year, respectively. The median frequency in groups B, C and D was 2, 3 and 3 respectively, showing again little to no location shift of the center of the frequency distribution in these statistics (Fig. 4). Despite the low average frequencies in these groups the proportion of cats in groups B, C and D considered to be dewormed in alignment with ESCCAP recommendations were 37.1%, 11.8% and 13.8%, respectively (Table 5). This is more positive than the results for dogs; however, due to the number of dewormings required to be considered aligned this may be an overestimate of the number aligned. Statistical analysis showed significant association between frequency of dosing and risk group (QCS = 13.8755, *df* = 1, *P* = 0.0002).

## Discussion

To the authors’ knowledge, this appears to be the first large-scale objective study in the UK examining deworming behaviour among owners of cats and/or dogs. It also appears to be the first survey in the UK to utilize owner information to assess risk factors that affect deworming frequency. Studies such as this are important to help assess if guideline advice is being accurately converted into effective preventative treatment protocols.

These survey results indicate that the majority of UK dogs (97%) fall within category D of the ESCCAP recommendations for deworming frequency on at least one risk-based criterion. Despite this, the mean and median doses were significantly below the 12 times a year deworming recommendation which is advised for this group. The very high percentage of dogs within high-risk Group D should be treated cautiously as the questionnaire respondents were not randomly selected which may result in response bias artificially affecting the results (e.g. owners most likely to respond to questionnaires might be in age groups which are more likely to live in rural areas, allow their dogs to roam further, have children, etc.). Respondents to surveys may also be more interested and involved in pet care and be more likely to follow vet or ESCCAP advice and deworm more often. It is therefore likely, on the basis of these survey results, that many dogs are at higher risk of parasitic infection and/or contact with groups at increased risk of zoonotic infections.

Although a lower percentage of cats were in high risk Group D (68%), the mean and median deworming frequency was also far below the 12 times a year frequency recommended for this group.

The lower percentage of cats in Group D compared to dogs is probably due to the relative ease with which cats can be housed strictly indoors. The data from this study also suggest cats are less likely to be in contact with children which may be due to inherent behavioural differences between dogs and cats.

A significant yet small proportion of cats and dogs were reportedly fed raw food. This trend is growing in Europe [21] and has the potential to infect cats and dogs with parasitic worms. Consumption of raw offal can lead to infection with tapeworms such as *T. hydatigena* and *E. granulosus* in dogs. Consumption of raw meat can similarly lead to infection with *Taenia* spp. and *Toxocara* spp. in both cats and dogs. Commercially available raw diets should have undergone meat inspection to human food standards and also should have been frozen to – 18 °C for 7 days or more in order to kill potential parasitic life stages. The freezing process is important, as there is the potential for parasitic life stages to be missed at meat inspection. Home prepped raw diets continue to have the potential to be fed, and dogs and cats still may be fed raw offal and/or meat from a number of sources which may have not undergone proper meat inspection, meaning that this route still needs to be considered in frequency of deworming.

It should be considered, when inferring that all dogs and cats should be dewormed 12 times a year that the final advice on deworming frequency must come from the individual risk assessment of the veterinary surgeon. Other factors such as environmental impact, unnecessary drug exposure of individual patients and anthelmintic drug resistance in helminths should also be taken into account, but not at the expense of minimising zoonotic risk and safeguarding patient health. Resistance has proven slow to develop in cats and dogs with large natural reservoirs of infection, when compared to equines and ruminants. Resistance is also only reduced by not treating infected cats and dogs with the potential for zoonotic life stages to be released into the environment. This makes deliberately withholding treatment to allow shedding of zoonotic worm eggs such as *Toxocara* and *Echinococcus* spp. difficult to justify on public health grounds. It may be deemed that pets are at low geographical risk for *E. granulosus* and *A. vasorum* infection or that children are of an age or hygiene education that precludes the need for increased deworming frequency. Veterinary professionals may also use frequent faecal diagnostic testing as an alternative to regular deworming as outlined in ESCCAP guideline 1. It would be useful for further studies to focus on specific risk factors and deworming frequencies in areas of Wales where *E. granulosus* is endemic. Even taking specific geographical and lifestyle factors not covered by this study into account, only 8.6% of dogs and 13.8% of cats met the deworming criteria for risk group D. Therefore, it is likely that a significant number of UK cats and dogs in group D are on inadequate deworming protocols in relation to endoparasite risk.

There is currently no evidence that deworming less than four times per year in dogs and cats has any benefit of reducing *Toxocara* spp. fecal egg shedding, and therefore no evidence of reduction of zoonotic risk [22–24]. This is of concern as the mean and median deworming frequencies for UK cats and dogs was below this. Given that 51.8% of cats and 80.6% of dogs had contact with children and/or the elderly, and especially with children being at particular risk of toxocarosis [10], this failure to adequately deworm represents a potentially likely and significant underestimated risk to health. There is, therefore, an opportunity to reduce risk of exposure by increasing the frequencies of deworming in UK high-risk groups. Eggs of *Toxocara* spp. which are passed in faeces are not immediately infective. It has been demonstrated that *Toxocara* spp. egg can embroyonate in the coats of dogs, but it is not at as high rate as in soil [25]. *Toxocara* eggs are long-lived in the environment and, without effective treatment of egg-shedding dogs and cats, numbers of infective eggs will increase in the environment.

Inadequate deworming programmes will also affect the health of cats and dogs, especially with worms which can be capable of causing potentially life-threatening conditions (i.e. *A. vasorum*). The low dosing average of dogs living in the South West and South East of England with a relatively high prevalence of *A. vasorum*, would suggest a significant number of dogs have been left unprotected. However, further work is required to ascertain whether these lower worming frequencies are due to an assessment of very local geographical and lifestyle factors or whether it represents a truly inadequate deworming frequency for this parasite.

Ensuring appropriate frequency of deworming is achieved after risk assessment is important in order to reduce risk of zoonosis and improve animal health and this study would suggest that in many cases adequate deworming frequency based upon risk assessment is not being achieved. Evidence, however, is lacking as to what point implementation of adequate deworming regimes currently failing. In order for appropriate deworming frequencies to be implemented: (i) veterinary professionals must first be convinced of the health benefits to both the public and to pets in order to implement effective risk-based parasite control programmes; (ii) veterinary professionals must also have access to lifestyle and current disease risk data to be able to advise appropriate treatment frequency; (iii) clients must be presented advice in a way that they can understand both how to implement effective deworming treatment and appreciate the added value and importance of implementing deworming programmes; and (iv) reminders for owners to give treatments at the correct time and frequency must then be provided.

Failure with any of the steps above will reduce treatment frequency. Research is required to establish in which steps failures are occurring to help ensure adequate frequency of deworming. In the meantime, the importance of conducting adequate risk assessments for all dogs and cats and prescribing adequate deworming based upon the ESCCAP guidelines should be emphasised to veterinary professionals. Effective methods aimed at improving treatment compliance among dog and cat owners such as apps, websites and social media in addition to practice care plans, should also be encouraged as tools to increase treatment compliance.

## Conclusions

This survey begins to address shortages of UK wide data regarding the lifestyles of pet dogs and cats in relation to their recommended deworming requirements and actual frequencies of treatments administered by pet owners. The large percentage of both cats and dogs in the highest risk groups suggests that the majority of dogs and cats should be on a monthly deworming programme due to at least one relevant risk factor being identified in the survey. Very few of cats and dogs in the survey met these deworming requirements. In order to reduce the zoonotic risk associated with *Toxocara* spp. and *E. granulosus* infection, pets with any risk of significant zoonotic egg-shedding should be dewormed at least four times a year. That the average treatment frequencies of pets in the survey population does not meet this recommendation is of major concern, given the large numbers of dogs and cats in contact with the elderly and/or young children. Further research is required to establish if these trends in inadequate deworming frequencies are genuine and whether geographical factors, diagnostics and detailed owner lifestyle information are affecting risk based preventative treatment advice. Additional research is also recommended to establish where failures in communication as well as application of adequate deworming frequencies are occurring. Simple, clear access to the recent data for veterinary professionals is vital to helping ensure they can evaluate risk accurately and provide effective advice to clients. Both veterinary professionals and the public need to be engaged in order to impress the importance of adequate deworming regimes, including aids given to pet owners which can help them remember when and how deworming should be administered. Veterinary professionals have responsibilities to conduct adequate risk assessments for all dogs and cats and while prescribing appropriate deworming based upon the evidence-based guidelines like those produced by ESCCAP. Only with veterinary professionals giving accurate advice in an easily accessible way, will treatment compliance and animal health be improved, and zoonotic risk may be decreased as a result.

## Data Availability

The datasets utilized in drawing the conclusions of this article are included in the article. Due to commercial confidentiality of this research, data not included in the manuscript can only be made available to *bona fide* researchers subject to a non-disclosure agreement.

## Abbreviation

ESCCAP: European Scientific Counsel Companion Animal Parasites

## References

[1] McNamara J, Drake J, Wiseman S, Wright I (2018). Survey of European pet owners quantifying endoparasitic infection risk and implications for deworming recommendations. Parasites Vectors.

[2] ESCAPP (European Scientifc Counsel Companion Animal Parasites). ESCCAP Guideline 01: Worm control in dogs and cats, 3rd edition; 2017. https://www.esccap.org/uploads/docs/0x0o7jda_ESCCAP_Guideline_01_Third_Edition_July_2017.pdf. Accessed 15 Mar 2019.

[3] Wilcox RS, Bowman DD, Barr SC, Euclid JM (2009). Intestinal obstruction caused by *Taenia taeniaeformis* infection in a cat. J Am Anim Hosp Assoc.

[4] Better Returns Programme (2015). Minimising carcase losses for better returns.

[5] Hydatid disease: making a comeback? Vet Rec. 2017;180:372. 10.1136/vr.j1814 28408509

[6] Nijsse R, Ploeger HW, Wagenaar JA, Mughini-Gras L (2015). *Toxocara canis* in household dogs: prevalence, risk factors and owners’ attitude towards deworming. Parasitol Res.

[7] Nijsse R, Ploeger HW, Wagenaar JA, Mughini-Gras L (2016). Prevalence and risk factors for patent *Toxocara* infections in cats and cat owners’ attitude towards deworming. Parasitol Res.

[8] Halsby K, Senyonjo L, Gupta S, Ladbury G, Suvari M, Chiodini P (2016). Epidemiology of toxocariasis in England and Wales. Zoonoses Public Health.

[9] Quattrocchi G, Nicoletti A, Marin B, Bruno E, Druet-Cabanac M, Preux PM (2012). Toxocariasis and epilepsy: systematic review and meta-analysis. PLoS Negl Trop Dis.

[10] Overgaauw PA, van Knapen F (2013). Veterinary and public health aspects of *Toxocara* spp. Vet Parasitol.

[11] Schantz PM, Weis PE, Pollard ZF, White MC (1980). Risk factors for toxocaral ocular larva migrans: a case–control study. Am J Public Health.

[12] Wells DL (2007). Public understanding of toxocariasis. Public Health.

[13] Wright I, Stafford K, Coles G (2016). The prevalence of intestinal nematodes in cats and dogs from Lancashire, north-west England. J Small Anim Pract.

[14] Morgan ER, Azam D, Pegler K (2013). Quantifying sources of environmental contamination with *Toxocara* spp. eggs. Vet Parasitol.

[15] Cobb MA, Fisher MA (1990). *Angiostrongylus vasorum*: transmission in south east England. Vet Rec.

[16] Morgan ER, Tomlinson A, Hunter S, Nichols T, Roberts E, Fox MT (2008). *Angiostrongylus vasorum* and *Eucoleus aerophilus* in foxes (*Vulpes vulpes*) in Great Britain. Vet Parasitol.

[17] Taylor CS, Garcia Gato R, Learmount J, Aziz NA, Montgomery C, Rose H (2015). Increased prevalence and geographic spread of the cardiopulmonary nematode *Angiostrongylus vasorum* in fox populations in Great Britain. Parasitology.

[18] Matos M, Alho AM, Owen SP, Nunes T, Madeira de Carvalho L (2015). Parasite control practices and public perception of parasitic diseases: a survey of dog and cat owners. Prev Vet Med.

[19] Office for National Statistics. Families and Households. https://www.ons.gov.uk/peoplepopulationandcommunity/birthsdeathsandmarriages/families/datasets/familiesandhouseholdsfamiliesandhouseholds. Accessed 15 June 2017.

[20] ESCCAP United Kingdom. Deworming frequency advice; 2017. https://www.esccapuk.org.uk/uploads/docs/q8e61ahf_003_Deworming_frequency_advice.pdf. Accessed 15 June 2017.

[21] Schlesinger DP, Joffe DL (2011). Raw food diets in companion animals: a critical review. Can Vet J.

[22] Traversa D (2012). Pet roundworms and hookworms: a continued need for global worming. Parasites Vectors.

[23] Beugnet F, Bourdeau F, Chalvet-Monfray K, Cozma V, Farkas R, Guillot J (2014). Parasites of domestic owned cats in Europe: co-infestations and risk factors. Parasites Vectors.

[24] Wolfe A, Wright I (2003). Human toxocariasis and direct contact with dogs. Vet Rec.

[25] Keegan JD, Holland CV (2012). A comparison of *Toxocara canis* embryonation under controlled conditions in soil and hair. J Helminthol.

